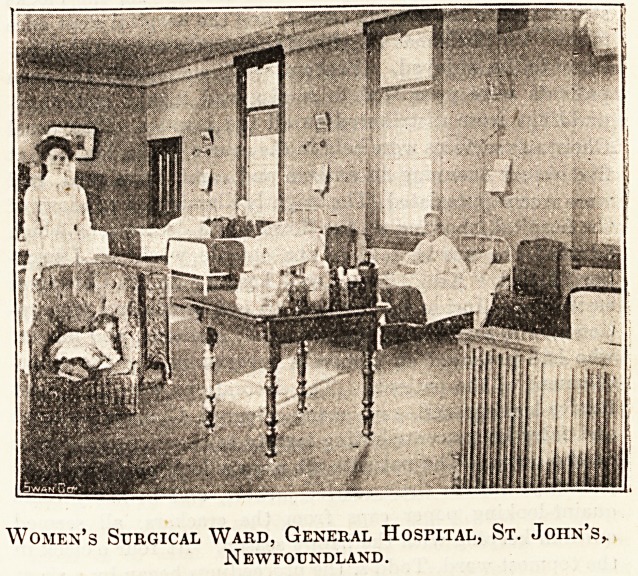# The Hospital. Nursing Section

**Published:** 1906-01-06

**Authors:** 


### The Hospital.

**Nursing Section.**

Contributions for "THE HOSPITAL," should be addressed to the EDITOR, "THE HOSPITAL" NURSING SECTION, 28 & 29 Southampton Street, Strand, London, W.C.

No. 1,006.—VOL. XXXIX. SATURDAY, JANUARY 6, 1906.

### Notes on News from the Nursing World.

### THE THREATENED STRIKE OF NURSES IN PARIS.

WE are informed, on the best authority, that the authorities do not regard the talk about a strike of nurses in Paris very seriously, and that, in fact, they doubt whether there will be a strike at all. Certainly they are satisfied that the nurses in training will not join in any movement of the kind. We hope in a subsequent number to publish an article explaining the precise position of the nurses in the Paris hospitals.

### NURSES AND DISPENSING.

HERE is an object lesson from the Antipodes showing the value to nurses in Australia of a knowledge of dispensing. The Committee of Gippsland Hospital, Sale, have just increased the salary of the female dispenser, who three months ago took the place of the male dispenser, from £50 to £75 a year. Board and residence are also provided. At the same meeting the Committee increased the salary of the matron from £70 to £84 a year. When it is added that the hospital at Sale contains a hundred beds, and no resident medical officer, the contrast between the salary considered sufficient for the performance of the matron's duties, which are practically ceaseless, and the salary paid to the dispenser, who is required to attend for three hours a day, will be recognised. We do not think that the matron in this case is adequately paid, but it is clear that in Australia, at any rate, nurses would do well to take up dispensing as post-graduate work, both because they would be able to increase their earnings and to considerably reduce the expenditure of the smaller hospitals. At home the supply of nurses who are qualified as dispensers probably exceeds the demand.

### STRUGGLES WITH LUNATICS.

A PRACTICAL proof has been given this week of the fact that nurses not only have to work hard, but occasionally run the risk of personal violence being done to them by patients under their care. In the Mater Misericordiæ Hospital, Dublin, on Sunday night, a male patient suddenly jumped out of bed, and on being spoken to by one of the nurses, turned upon her and stabbed her with a penknife which he had previously taken from a locker near his bed. A doctor and a student, hearing the cries of the nurse, rushed to her assistance, and after a most exciting struggle, in the course of which the student received a stab in the leg, the patient was secured. He was subsequently charged in the police court, and after medical evidence had been heard, he was committed to a lunatic asylum. In another case reported this week, at Tynemouth Workhouse Hospital a male attendant was attacked by a lunatic who had been placed in a padded room. The attendant, relaxing his vigilance for a moment, the patient sprang at him and dealt him a violent blow in the face, breaking his nose. In the end the lunatic was overpowered, and he has since been conveyed to the County Asylum at Morpeth; but the attendant, in addition to facial injuries inflicted, is suffering from the shock. Possibly the second of these savage attacks might have been guarded against, but the Dublin nurse could not, by any attention or forethought of her own, have anticipated the assault made upon her. We hope that neither of the victims will suffer any permanent ill results from their untoward experiences.

### THE VALUE OF THE ORDER OF 1897.

AN extraordinary attempt was made at the last meeting of the Chelmsford Guardians to put back the clock. Under the provisions of the Nursing Order of 1897, the medical officer of a workhouse has power to engage a temporary nurse. This power was recently exercised by Dr. Thresh at Chelmsford because one of the out-poor had been attacked with measles. His action was unfavourably criticised by several of the Guardians, who contended that, instead of paying a temporary nurse £2 a week, a member of the Infirmary staff should have undertaken the charge of the child suffering from measles, and it was even proposed to censure him. But on the other side it was urged that the seven nurses working in the wards were fully occupied, and that in any circumstances it would be exceedingly dangerous for one of the in-door staff to attend an infectious case and go backwards and forwards to the Infirmary. The chairman, having stated that a case of small-pox in the workhouse once cost the Guardians £400, declined to put a motion for a vote of censure on the medical officer. We may add that the latter, in discharging his duty, took the best possible course, both for protecting the children in the workhouse, of whom there are between 40 and 50, from measles, and for saving the pockets of the ratepayers.

### THE ADMISSION AGE IN NEWFOUNDLAND.

IT will be seen from the interesting illustrated contribution on the nursing at the General Hospital, St. John's, Newfoundland, which appears in another page, that the admission age in that distant part of the King's dominions is 21. The training at the hospital in St. John's is for three years; but as the school was only started about two years ago, it is not yet possible to judge the effect of a rule which we think would not be a suitable one in the Mother

Country. In Newfoundland, where girls mature more quickly, the policy may be sound, especially if, as it would appear, those who desire to take up nursing are generally under 21. The account which our contributor gives of the nurses returning from sliding with rosy cheeks, looking pictures of health, suggests that, at any rate, their physique does not suffer from the strain of their duties in the wards.

### TROUBLE AT HULL SANATORIUM.

A short time ago the lady superintendent of the Hull Sanatorium complained that the nurses under her control had been insubordinate, some of them adding insolence to disobedience. A sub-committee was therefore appointed by the Hull Sanitary Committee to inquire into the matter, and their report has just been discussed. They found that there had not only been friction between the lady superintendent and the nursing staff, but also between the staff themselves. They had not been able to discover that the patients had suffered, but as they hold that it was of the highest importance that the officials should work amicably together, they recommended that the chairman should caution the sister in charge of the diphtheria ward; that a nurse who had refused to obey orders, and was about to take her annual holiday, should be asked not to return to the sanatorium; and that another nurse, who had been telling tales, and on her own initiative had mutilated a report book, should be requested to resign. These recommendations were adopted by the Sanitary Committee, and the lady superintendent will accordingly have another opportunity of showing whether she can choose suitable nurses and maintain discipline amongst them when she has got them.

### BOGUS NURSES AS COLLECTORS.

It is seldom that even the much-abused uniform of a nurse has been put to such a disgraceful use as at Liverpool. In that city several women attired as nurses have been making collections for charities which, on investigation, proved to be impositions. We fear that there is nothing to prevent unauthorised individuals from masquerading as nurses, but when persons attired in nurses' costume endeavour to collect money they should be asked the name and address of the institution to which they belong. If the public will only exercise the most ordinary care, they can at least protect themselves from fraud by women who imagine that the mere adoption of a nurse's uniform will enable them to succeed in nefarious purposes.

### POOR-LAW NURSES AND OUTSIDE WORK.

In the report of Mr. Baldwyn Fleming, Inspector for Dorset, Hampshire, and parts of Wiltshire and Surrey, which forms a portion of the report of the Local Government Board for the year 1904-5, reference is made to the question of nursing the out-poor. Mr. Fleming thinks that it would be a useful experiment if a Board of Guardians could try to arrange for nursing them by the indoor nursing staff. Since he expressed this view the Croydon Guardians, as we intimated last week, have fully considered the advisability of making the experiment which he advocated, and found that it was not desirable to attempt it. Mr. Fleming confesses that the workhouse staff would have to be increased, and that the elements of distance and locomotion might limit the application of the system in some places. But he contends that where local circumstances are favourable the plan would present considerable advantages; and he bases his contention chiefly upon the ground that the out-door work would give most valuable experience to the nurses, as well as relieve the monotony and routine of their in-door duty. We recognise the force of these pleas, but we adhere, nevertheless, to the view that it is better that the nursing of the poor outside the workhouse should, for the most part, be left to qualified district nurses. We are glad to see that Mr. Fleming favours the contributions by Guardians to District Nursing Associations, and in this direction we think with him that more liberality might be shown.

### PROPOSED WITHDRAWAL OF EAST LONDON NURSES.

We regret to learn that the Committee of the East London Nursing Society are seriously considering the question of decreasing their staff of nurses. They would be most reluctant to take this step, and it will only be forced upon them if the further help, for which an appeal has been made to the public, is not speedily forthcoming. The cost of each nurse is between £60 and £70 per annum, all the nurses employed being fully trained. The organisation is most admirably managed as well as staffed, is undoubtedly one of the most beneficent associations in the East End, and, instead of any nurses being withdrawn where they are so urgently wanted, we should like to see their number increased.

### ALLEGED FRICTION AT A WORKHOUSE INFIRMARY.

It having been stated by a former assistant nurse at Stratton Workhouse Infirmary that she had resigned in consequence of her inability to work under the superintendent nurse, a committee of the Swindon and Highworth Board of Guardians inquired into the matter, and came unanimously to the decision that there was no ground for the complaints against the superintendent nurse. The chairman, who was himself one of the committee, and other speakers, mentioned that the rest of the nurses stated that they had no grievance against the superintendent, but the report of the committee was not adopted without protests on the part of several Guardians, who seemed to think that the late assistant nurse had some cause for dissatisfaction. The members of the nursing staff, however, affirm that she talked of resigning her post for two years, her reasons being dislike of night duty and insufficient amusement in off-duty time.

### THE MILITARY NURSING SERVICE.

We are officially informed that Miss L. M. Moor, sister in Queen Alexandra's Imperial Military Nursing Service, has been transferred from the Royal Arsenal, Woolwich, to the Military Hospital, Chatham. Miss E. B. Darnell, staff nurse, has been transferred to the Royal Arsenal, Woolwich, on her appointment. Miss M. E. Neville, staff nurse, has been posted to Malta on expiration of her sick leave. Miss A. E. Tait, matron, has resigned her appointment, and has been granted permission to retain the badge of Queen Alexandra's Imperial Military

Nursing Service, in recognition of her meritorious service.

### THE NEW MATRON OF HAMMERSMITH POOR-LAW INFIRMARY.

The appointment of Miss Eve Ward as matron of the new Hammersmith Poor-law Infirmary is notable, if only because it is one of the rather rare instances of a plum of the profession falling to the lot of a Poor-law trained nurse. Miss Ward was trained at the Hope Hospital, Salford, where she afterwards became sister and acted temporarily as night superintendent. During the last four years she has been assistant matron at St. Luke's Hospital, Halifax. As she went to St. Luke's Hospital almost directly after it was opened, she has enjoyed the kind of experience which she will find particularly useful in the new institution at Wormwood Scrubs. She has not only helped in all departments of administrative work at Halifax, but for some months she took matron's duties, so that she quite understands the requirements of the position for which she has been selected. The choice of the Hammersmith Guardians will, doubtless, be an encouragement to Poor-law trained nurses who are anxious to become matrons in their turn.

### NURSES' ENTERTAINMENT AT GUY'S HOSPITAL.

On Friday last the nurses at Guy's Hospital had their Christmas entertainment, at which the residents of the medical staff were guests, also Sir Cooper and Lady Perry and Mr. and Mrs. Cosmo Bonsor. The home was beautifully decorated, and made into a sort of fairy palace for the time being. The large dining-hall was used for the entertainment, and at one end a capital stage was erected. Refreshments were afterwards served in the general sitting-room and adjoining rooms. The programme was an unusually good one, both nurses and sisters having worked hard to produce excellent results. From 8 p.m. until 9.30 p.m. the night nurses were responsible for their guests' entertainment, after which time they went on duty, leaving the stage clear for the day nurses to display their powers of elocution in play and song. Some of the items were quite original, portraying various well known characters and scenes in the hospital. The surgical tableau, "At the Front," was encored again and again, as was also the caricature of a "scrubber" of 30 years' standing. The very pleasant evening ended with an hour's dancing in the dining-hall, and the party finally broke up with the singing of "Auld Lang Syne," followed by three cheers for the matron.

### A NEW DEPARTURE AT PHILADELPHIA HOSPITAL.

A special course in the nursing of tuberculosis has been inaugurated at the Philadelphia Hospital. It is intended to afford an opportunity for self-support and a useful career to women who have had tuberculosis and to any others to whom the work may appeal. The length of the course is two years, and those who wish to take it must be at least twenty years of age and free from physical defects other than tuberculosis. The nurses will only be required to remain on duty for eight hours each day, but they will be expected to spend two additional hours in study. They will receive their practical training in the tubercular wards of the Philadelphia Hospital, and a special course of lectures will be given them on the care of tuberculous patients.

### THE RIVIERA NURSES' CO-OPERATION.

Considerable interest has been excited by our intimation that a Nurses' Co-operation has been started in Nice. We now learn that it has been decided to call the organisation the Riviera Nurses' Co-operation, and we are asked to state that nurses who desire to join must not be entirely dependent upon what they earn, and must have good training and experience in private nursing. We may add that the weekly cost of living, when a nurse is not at a case, is £1 2s. 6d. and that any other particulars may be obtained from Miss Florence Warcup, 18 Boulevard Victor Hugo, Nice.

### EMERGENCY VILLAGE NURSES.

The annual meeting of the Sussex County Nursing Association was held at the London residence of Lord Brassey, who himself moved the adoption of the report, the chair being taken by Mr. W. D. James. The importance of the emergency nurses was dwelt upon by several speakers. These are the nurses who are trained for nine months under the auspices of the Association; and, as a speaker at the meeting put it, when the villages seeking affiliation ask what they will get out of the Association, the answer can be given in three words, "the emergency nurse." There are now thirty local organisations in the county, covering sixty-six parishes and employing forty-three nurses. The financial statement also shows a balance on the right side, and altogether it is not only performing useful functions but it is managed on sound business principles.

### PIONEER NURSING IN THE HIGHLANDS.

For three years the duties of district nurse in Park, Stornoway, have been discharged by Miss Macritchie who has just resigned in consequence of her marriage. Located at Lemreway, she has tramped over the moors in all sorts of weather with unruffled cheerfulness whenever she was wanted, and has made the local nursing association a power for good in the Highlands. Her relations with the people among whom she laboured, the medical men in Stornoway, and the Committee of the organisation under whom she worked, were of the happiest kind; and before her departure a minute of thanks for her services and congratulations upon her approaching marriage, was presented to her. It is hoped that a successor will be secured who will show the same devotion and ability which she manifested in a post that, if still arduous, has been exalted by her efforts to one of real influence for good in a variety of ways.

### SHORT ITEMS.

At Workington, Cumberland, a new home for the nurses employed in outside work has just been opened.—A community of nuns of British birth, known as the Little Company of Mary, are opening, at Rome, with the Pope's approval, a home in which only English-speaking patients will be received.—The new assistant nurse of Malton, Norton, and District Cottage Hospital is Miss Mable Hartley.

### The Nursing Outlook.

"From magnanimity, all fear above;
From nobler recompense, above applause,
Which owes to man's short outlook all its charm."

### TWO HOMELY VIRTUES.

HONOUR and bravery, truth and candour, love and unselfishness, how well they go together, and what echoes they call up in our minds of many a hero and heroine, some well known to fame, and others that we have just come across in our own little experience. But thrift and temperance!

Thrift? If there are two things that the average young person believes, they are that she will never grow old, and that none of the old persons she knows can ever have been young. Then, she thinks, why lay up for a day that is never coming?

Certainly it is a day to put off as long as possible. Some famous man said that it is better to be 70 years young than 30 years old. But though we must be old in years, if we live long enough, we can try to keep ourselves as young in heart as possible; and surely one way of attaining this is by not allowing ourselves to worry. And if we know, that in consequence of our thrifty ways, we are laying up for ourselves some provision for our old age, we shall certainly be spared many a dismally foreboding moment, many a worrying anticipation.

It is a libel on the virtue of thrift to suppose that it is the same thing as niggardliness. It is the thrifty who can afford to help other people; not those whose so-called generosity flings about their money with a blind recklessness of what the future may bring. If one be assured of a certain provision for one's old age, it is much easier to spare a little now to help a lame dog over a stile. Indeed, many people find that their money goes just as far, and with much more satisfaction, if a tenth be deducted from it when it is received, and put away in a special purse for the service of the poor.

Although we are not writing on behalf of any particular savings bank, or insurance society, every wage-earning woman should put regularly into one or the other. The Royal National Pension Fund has many advantages for a nurse: to begin with, the managers have so much to do with nurses that they are able to give them very useful advice as to the manner of insuring or saving. Then, though it is easy to withdraw money that has been paid in, it is less fatally easy to do so than from the Post Office Savings Bank, and this small amount of delay often results in the money being left on deposit for some time of greater need instead of its being hastily withdrawn.

Moreover, as the premiums on an annuity have to be paid at regular intervals, there is not the same temptation to say, "Oh, I can't spare three pounds this month; I will put away an extra sovereign next time," as we all know is the case when the money may be saved at our own discretion.

It is a pity to forget that every pound we put away this year will bring us in more when we really want it than if we leave it till next year before putting it away; and that if we join the Pension Fund this month we shall pay less for the same amount in our older days than if we do not begin to save till next year.

Then as to the other homely virtue. The old rhyme says, with a lamentable want of politeness:—

"Joy and temperance and repose,
Slam the door on the doctor's nose."

It is not to be supposed, however, that the poet's view of temperance was the limited one which confines the word to the moderate use of food and drink; certainly not the modern usage of temperance for "total abstinence from intoxicants." But it is from that side of temperance that we want to regard the matter from a nurse's point of view.

It is unfortunate that some better word is not found to express this habit of total abstinence than "teetotalism." The thing itself would be less unpopular if it were not called by such a meaningless epithet.

For, though temperance is a virtue, teetotalism as such is not one. It is no credit to the baby at the breast nor to the prisoner in gaol that he is a teetotaller. But when it is practised as a self-denial for the sake of others, surely then it is lifted somehow to a higher plane.

It comes very near to a necessary virtue when a nurse finds herself working in the midst of people with whom excess in alcohol is frequent, if not habitual. Every nurse knows that most wards in her hospital would have a very empty look if all the victims of alcohol in some form or other were weeded out. Every district nurse sees that one of the chief barriers to even the poorest leading clean and self-respecting lives is their habit of spending far too large a proportion of the week's earnings in intoxicants. And in the houses of the well-to-do, in spite of "three-bottle men" having departed with the change in national habits, the private nurse sees that many of her patients are suffering, directly or indirectly, from alcoholism.

Only those who have done the work know how much inducement there is to the private nurse to drink. We do not say temptation, for it is not a temptation to one in a hundred; but there is the perpetual invitation to drink. It continually happens that the first greeting at a new house is, "Won't you have a glass of something before you go upstairs?" and a "little drop of brandy, after such a nasty job" was a daily suggestion to a young nurse after finishing a dressing that had to be repeated several times a day. Two nurses, in charge of a severe case of pneumonia in a suburban hotel, found a bottle of whisky in the bedroom of the elder, but, as they remarked afterwards, "Nurse So-and-So was put off with a bottle of port; I suppose they thought her a little too young for spirits!"

There is this to be said for teetotalism, that it is an easy habit to cultivate. And if it happens to anyone that it is harder to give up the daily stimulant than even the almost necessary cup of tea, perhaps it is time to rank total abstinence among the virtues!

### Abdominal Surgery.

By Harold Burrows, M.B., F.R.C.S., Assistant Surgeon to the Bolingbroke Hospital.

### PERITONITIS.

Peritonitis is the name given to any inflammation of the lining membrane of the abdominal cavity. Cases may be divided into three main classes, as follows:—(1) Simple, (2) Toxic, (3) Infective.

*Simple peritonitis.*—A mild inflammatory reaction is produced by any local injury, and the process may be watched during a laparotomy in which viscera are exposed. When the abdomen is opened, the peritoneum covering the coils of intestine is at first pale, glistening, and slippery. As time goes by, it becomes reddened, the surface loses some of its shine, and instead of being slippery becomes sticky, so that neighbouring coils of intestine adhere to each other; and if abdominal sponges have been used it will be noticed, when they are being withdrawn towards the end of the operation, that the intestines adhere slightly to them. The reddening, loss of polish, and stickiness are the results of inflammation. This so-called simple peritonitis rapidly clears up after the abdominal wound has been closed, and there is little or nothing of practical value to say about it, except that it should be prevented, so far as possible, by having the lotions at a proper temperature (about 105° F.) and by taking care that they are not composed of unduly strong solutions of irritating antiseptics.

*Toxic peritonitis* is chronic in its course, and is caused by the circulation of certain poisons in the blood, as may occur in disease of the kidneys, for example. The simple and toxic forms are relatively unimportant so far as the purposes of this article are concerned, and therefore they will not receive any further consideration.

*Infective peritonitis.*—In common usage the term "peritonitis" means an inflammation due to the presence and growth of micro-organisms in the abdominal cavity. Such inflammation may be *acute* or *chronic* in its course; it may involve a limited portion only of the peritoneal cavity, when it is described as *local* or *localised*; or it may spread far and wide, and so become *diffuse* or *general*. Peritonitis may be qualified, also, in terms of the causative agent; for example, *tuberculous*, *pneumococcal*, *gonococcal* peritonitis, which are due to the tubercle bacillus, the pneumococcus, the gonococcus respectively.

### Paths of Infection.

There are several ways by which micro-organisms may gain access to the abdominal cavity. The alimentary canal is teeming with virulent germs, and any injury or disease of its walls may allow some of these germs to pass through and infect the peritoneum. This is especially liable to occur in connection with the vermiform appendix, which is a frequent seat of disease. Other sources of infection are the liver and gall-bladder, the uterus and uterine appendages, the urinary organs (bladder, ureters, kidneys), accidental and operative wounds of the abdomen; or the micro-organisms may be conveyed to the peritoneum and deposited there by the blood-stream. By whatever route the infection has travelled its consequences will depend on the quantity of germs introduced, on their virulence, and on the patient's powers of resistance. Occasionally the germs may be destroyed without causing more than a very slight and transient peritonitis, or they may establish a local peritonitis with the formation of an abscess, or they may quickly overcome all opposition and bring about fatal diffuse peritonitis. The means which nature employs to resist bacterial invasions in this region are two-fold. In the first place, the patient's blood is endowed with a variable power to destroy the germs of disease; and secondly, by adhering together, and to the parietal peritoneum, the abdominal viscera prevent the invading organisms from spreading. From this it will be seen that the inflammatory reaction known and dreaded as peritonitis is really protective in character. The reddening of the peritoneum is due to dilatation of its blood-vessels by which as much blood as possible is enabled to reach the field of battle, so to speak, and the stickiness of the peritoneum is the means whereby the infection is prevented from becoming general, for the abdominal viscera adhere to each other and to the abdominal wall, and so help to confine the infection to a limited area.

As local peritonitis will be considered in connection with the various organs from which it may originate, I will confine my remarks here to acute diffuse peritonitis.

### Acute Diffuse Peritonitis.

*Symptoms.*—Severe abdominal pain is the predominant symptom in most cases. It may come on suddenly, as in perforation of a gastric ulcer; or the pain may be mild at first and gradually increase in severity. Soon after the onset of pain the patient begins to retch and vomit, and has to lie down. He lies on his back, with his knees flexed, and his expression soon denotes intense anxiety ("abdominal expression"). On feeling the pulse it is found to be small in volume, hard, and increased in frequency. The temperature is probably raised, though it may be actually lowered—and it may be mentioned here that an unduly frequent pulse in conjunction with a subnormal temperature is a sign of ill omen in a case of peritonitis. The patient begins to complain greatly of thirst, and shock becomes well marked. The abdomen is very tender everywhere, is hard, and does not move with respiration. If the disease is allowed to pursue its course the patient rapidly goes down hill. The pulse becomes soft, and is at last impalpable, the abdomen becomes distended, the tongue becomes foul, and the lips dry; the vomit, which at first consisted of the ordinary contents of the stomach, becomes bilious, and later brown and stercoraceous, and at last the vomiting may give place to hiccough, and the patient dies.

This is the general picture of a case of fatal acute diffuse peritonitis, but the details are not the same in all cases, and too much reliance must not be placed on individual symptoms; it is the aggregate that is rather to be considered.

The duration of a fatal case is variable. The patient may succumb within thirty-six hours of the onset, or may linger on for a week or more.

The main difficulties in diagnosis are to distinguish general peritonitis from local peritonitis, from colic, and from certain other illnesses accompanied by general abdominal pain. But as a suspicion of general peritonitis is an imperative reason for instantly seeking efficient surgical aid, the minutiæ of differential diagnosis will not be discussed here.

*Treatment.*—There is only one method of treatment which offers any material hope of saving the patient's life, and that is immediate operation. A large proportion of cases end in recovery if operated upon within twelve hours of the onset, but very few recover if operation is delayed beyond 24 hours. So that if a patient is thought to have general peritonitis, surgical assistance should be obtained at once and preparations made for the expected operation. With regard to the treatment of the patient meanwhile, there is not much to be done beyond keeping him absolutely at rest. A pillow should be placed under his knees, and a cradle will be necessary to relieve him of the weight of the bedclothes, and hot fomentations applied to the abdomen may relieve his pain somewhat. These fomentations should be wrung out of 1-1,000 biniodide or perchloride of mercury so as to act as a preparatory dressing. As the abdomen in these cases is extremely tender, it is not possible to prepare the skin thoroughly until the patient is under an anæsthetic. In the meantime nothing should be given by the mouth, and no medicines, not even brandy, should be administered. This course may appear at first sight to be cruel, because by giving opium the intense pain can be greatly relieved; but there is this danger, that by preventing the pain and vomiting and other symptoms opium conceals the gravity of the case, and therefore frequently leads to fatal postponement of the only curative measure, namely, operation. The objects of operating in these cases are to remedy, if possible, the defect which has permitted the escape of micro-organisms into the peritoneal cavity, to remove by mopping or by flushing out the abdomen with large quantities of sterilised water or normal saline solution the germs which are already present, and to provide for the escape of the products of septic inflammation by drainage. Therefore in making the arrangements for an operation on a case of septic peritonitis, an irrigator with large quantities of sterilised normal saline solution should be prepared, and large-sized drainage tubes provided, in addition to the instruments required for performing a laparotomy.

### The Nurses' Clinic.

### THE DISTRICT NURSE IN RELATION TO THE TREATMENT OF HIP DISEASE. BY MISS M. LOANE.

THERE are few cases in which the district nurse's services are of more value than in relation to hip disease; in the first place because hip disease is intensely painful, and she can do much to alleviate the suffering caused by it; in the second place because it is a complaint which, as the poor express it, "makes a heap o' work," work which under skilled direction can be organised and simplified until the previously overburdened mother has far less to do, and the child is far more benefited by what she does; and thirdly because the surroundings which have induced one instance of hip disease are likely to induce others, and the nurse will have numerous opportunities of detecting symptoms in the neighbours' children during the early and more hopeful stages.

Unfortunately, it rarely happens that a district nurse gets a case of hip disease on her books until that particular sufferer is in a practically hopeless condition, but I recently had a case which was well on its course before we heard of it, but which has nevertheless made satisfactory progress and shows every prospect of cure. The patient was a little girl of two and a half, the only child of poor but very respectable parents. The illness had developed some months before and a doctor had been in attendance, but owing to mismanagement of the nursing part of the work the child was in a terrible state. I attended her occasionally for a short time, but the expense and fatigue of keeping her at home any longer—the direct expense had already reached £30—proved too much for the mother, and the child was sent to hospital for three months. She returned home in much better condition, and as she had been brought into some kind of training and taught to ask for all she wanted, and as the mother was now rested and in a hopeful frame of mind, the opportunity was seized to make a thorough reform of all the nursing treatment.

The child had previously been kept chiefly in the kitchen, which, though not dark, was gloomy, and received no direct sunlight. The mother was now induced to remove the bed to the front sitting-room, which had a sunny aspect, and to allow the blind to be kept up and the window-sash to be lowered, whenever the weather permitted. The bed was raised so as to be on a level with the window, and a piece of wood was placed across it to serve as a table. This arrangement gave the child light, air, and a comparatively cheerful and interesting prospect, and relieved the mother from the fatigue of continually stooping whilst attending to her. The mattress was made perfectly smooth and flat, the under-sheet and draw-sheet were firmly fastened to the edge with safety pins, and the mother was warned to keep the bed free from crumbs, to keep the bedding absolutely dry, to wash the child's back with soap and water twice a day, and then to rub it with methylated spirit and powder, dipping her finger in powder in preference to using a puff. Powder put on thickly is apt to cake and cause more irritation than it relieves. Only one small pillow was allowed. A bed-pan was made by fitting a rim of wood to a round tin dish.

An extension was then fixed, and the weight ordered by the doctor was attached. This weight is proportioned to the child's size and strength and other circumstances, and it is a point as to which the doctor's special instructions must always be obtained. Eight strips of brown holland strapping 16 inches by 1 inch were needed, and two straps 8 inches by 3 inches; the stirrup was made of a piece of wood 4 inches by 2 inches with a hole cut in the middle; a large knitting needle, a large empty silk reel, half a yard of blind cord, an improvised cradle to keep the weight of the bed-clothes off the great toe, and a padded toe and heel cap, completed an effective and inexpensive extension apparatus.

The mother was taught, further, that complete rest of the joint was absolutely necessary, that she must always have the sound limb next her when lifting the child, and keep the injured one perfectly straight, that she must avoid pushing against the weight of the extension or jerking the child in any way, and that she must always lift the weight when giving the bed pan.

I explained to both parents how essential it was that the child should be taken out into the fresh air every day, but that it must be done without altering the rigidity of her position. The father, a most intelligent man, and passionately devoted to his little daughter, obtained an illustrated catalogue of invalid carriages, and after diligent study of it and much labour, produced, at a cost of 8s., a perambulator which answered the purpose as well as the most expensive on the list would have done. The body of the carriage was a flat basket of the right length and width, four wheels from a discarded go-cart, and convenient handles were fitted to it, and a light awning was arranged to save the child's eyes from the glare. Every day the mother wheeled her out, choosing the airiest, sunniest spots accessible, walking slowly and passing cautiously from one level to another.

Even at the worst point of its sufferings this child had always shown the hopeful sign of not losing flesh, and under the strict treatment described above the pain and swelling gradually reduced, the terrible sores healed up, and the child, now four years old, is able to use a Thomas's splint, and will probably make such a complete recovery that even the shortening of the leg will be almost imperceptible.

When sores exist they should be treated like all wounds of that nature, but if there is much pain the outer dressings should be four-fold linen, 6 in. wide, in preference to narrow bandages, which cannot be applied without frequently lifting the limb.

It must be remembered that hip disease commonly originates in children of a tuberculous tendency who have received some injury to the leg. Parents should be warned to pay heed to all complaints of pain in the joints, and to all awkward and unusual movements in walking, especially a tendency to walk on the toes of one foot while the other is used in the ordinary way. There are symptoms that ought to be visible to an observant mother long before the disease has gone so far that the child wakes in the night with a scream of pain. In the very early stages complete rest would probably prevent the formation of abscesses and all their attendant pain, exhaustion, and misery.

A nourishing diet is required, but the child must not be fed at too frequent intervals, and some gentle check must be put upon the continual presents of cakes and sweets and nuts brought by pitying fathers and uncles. The money should rather be spent on toys and picture-books, or saved for some expensive necessary.

### Presentations.

Bradford City Hospital.—Miss Mellor, of the City Hospital, Bradford, was presented, on December 29, by the staff, with a handsome silver fruit-dish, suitably inscribed, on the completion of twenty-one years as matron. The Chairman of the Health Committee was present, along with the staff and a large number of friends.

Loughborough Hospital.—Last week a presentation was made to Miss Baker, who is leaving Loughborough Hospital after over fifteen years as matron of the institution. The gift was initiated by the lady visitors, and the invitation to participate was readily responded to, with the result that a sum of £45 was obtained. The Mayor of Loughborough, in handing the purse to the recipient, spoke of her long service to the institution. He said that he was very pleased to comply with the request of the lady visitors to make the presentation, for he had heard so much in the town of Miss Baker's useful work and of the high esteem in which she was held. Miss Baker acknowledged the gift with expressions of gratitude to the subscribers and lady visitors.

### Incidents in a Nurse's Life.

Contributions to this column are invited.

### A STEAM TENT UNDER DIFFICULTIES.

A card was sent to me one winter's morning desiring me to attend the case of a boy who had scalded his throat through drinking hot water out of a kettle. The doctor ordered a steam kettle and tent, neither of which appliances I possessed.

When I arrived at the house, which was healthily situated in a blind alley, I found the little patient, aged five, in the general living room, which was full of children; I counted eight, the eldest about fourteen, the youngest a baby in arms; the mother with her face tied up; the grandmother and the great-grandmother also being present. The room was about twelve feet by ten. The table was crammed with dirty crockery, the patient was huddled up on a very dirty leather-covered sofa under the window, surrounded by a general assortment of rags, old coats and shawls and a pillow. When I saw the child, I thought he would only live a few hours; his breathing was stertorous, and he appeared to be quite unconscious. The first thing I did was to fetch a steam kettle from the chemist's—fortunately close by—and to put it on; then I made the child as comfortable on the couch as I could, with a pillow or two, a coloured blanket round him, and the best of the shawls. I drew the couch forward to the fire, displacing a few children who were sitting on the floor. I asked the mother for two long sticks and a high clothes-horse. I tied the two sticks to the legs of the sofa under the head, and the clothes-horse I tied to the legs at the foot. I fastened a sheet over this erection the best way I could. I gave the boy a little milk with a spoon during the time I was there—nothing had been given previously—and told the mother to continue it, thinking, however, that it was of little use. I asked her if she had another room, as that one was not fit for him to be in. She took me into the "best parlour," which smelt damp and musty; antimacassars on every chair, and the best dinner service set out on the cupboard. The room had a fireplace on the right-hand side, opposite to which was a bay window; there were low cupboards in the recesses at the sides of the fireplace. I saw I could have the couch head against one of these, and I asked the mother to nail up two pieces of wood against the front of the cupboard for supports for the tent. I told her she must light a fire at once, and that I would come again in a few hours' time. On my second visit, I did not expect to find the child alive; to my great surprise he was a little better. The front parlour was nicely aired. I set to work and rigged up a tent with sheets, putting a sheet over the two pieces of wood, so that it fell down to the top of the cupboard. I nailed it along the wall inside the tent, so that it hung down in a flap that side. I had a high clothes-horse where the foot of the couch would come; the sheet hung down over this to the floor. I tied a piece of rope from the top of the wood nailed to the cupboard to the top of the clothes-horse, and the sheet hung down over this. I pinned a small sheet down the corner of the clothes-horse and along the rope, to form a curtain. Then the mother and I carried in the couch with the child upon it, and placed it in the tent. I did all that was necessary for him, and told the mother to keep the steam kettle going all night. The next morning I went again. He was still a little better; at each succeeding visit there was a gradual improvement; and I was able to leave him in less than a fortnight, perfectly recovered.

### Across the Seas: Nursing in Newfoundland.

THE General Hospital, St. John's, Newfoundland, stands on the side of Signal Hill, overlooking the town and just outside the city limits. A better site could hardly be found. It is away from the city, though within a few minutes' walk, it is sheltered from the sea by the hill, but gets the full benefit of its health-giving breezes. The grounds are fairly large, and are now being newly fenced and laid out. The front of the hospital looks towards the town, the back looks out on as pretty a bit of autumn scenery as one could find anywhere: rugged brown rocks, dark green fir-trees—green all the year round—and the beautiful red of the whortleberry bushes.

GENERAL HOSPITAL, ST. JOHN'S, NEWFOUNDLAND.

The exposed position of the hospital in winter causes the snow to lie in very deep drifts, and at one time last winter the inmates were practically snowed in for three days. Telephone and electric wires were broken down by the heavy snowstorm, and the hospital was perfectly shut off from the outside world. The staff had to utilise all the kerosene lamps and candles which could be procured to give the necessary light. The drifts were so deep that it was impossible to get over them except on snow-shoes.

NURSING SUPERINTENDENT'S ROOM, GENERAL HOSPITAL, ST. JOHN'S, NEWFOUNDLAND.

To the north of the hospital is Quidi Vidi Lake, where the boat-races are held in August. The day is always a public holiday, and even in hospital it is as much a holiday as it can be made. All who can, go out into the grounds, and all others in bed who are at all well enough have their beds wheeled to the windows, and are propped up so as to see the races; and the patient who is not interested on this occasion must be very ill indeed. They are generally provided with programmes, and the excitement in the wards is almost as great as that around the lake. There is no nursing home—the nurses have to sleep in the hospital—but it is hoped that very soon this state of things will be remedied. The whole hospital is much cramped for room in every way, but in 1906 a new wing for sixty patients is to be added, with laundry, operating theatre, and accommodation for nurses. The building is heated with hot water; on very cold days in winter sometimes there are fires as well. It is lighted by electricity, a light being placed over every bed, and in the operating theatre there are electric heaters for warming the table.

MEN'S SURGICAL WARD, GENERAL HOSPITAL, ST. JOHN'S, NEWFOUNDLAND.

WOMEN'S SURGICAL WARD, GENERAL HOSPITAL, ST. JOHN'S, NEWFOUNDLAND.

The nursing staff consists of sixteen probationers. None of the nurses are fully trained, as the training school was only started about two years ago. Two of the original nurses who were at the hospital when the school started still remain. The work of reorganisation has perforce been done slowly, and as the old nurses dropped off, their places were filled by new probationers, more suitable for training. The probationers come for a month on trial, and if accepted sign an agreement for three years. They are paid $48 the first year, $72 the second, and $100 the third year. A certain amount of uniform is given them after the first year—out-door uniform is not worn. The age for admission is from twenty-one to thirty. By far the greater number of applicants are under twenty-one.

The nurses are called at 6 A.M., breakfast at 6.30, and go on duty at 7. At 9 and 9.45 they have three-quarters of an hour for lunch, bed-making, &c. They have one hour and three hours off duty on alternate days. The one hour is taken in the morning with the lunch time, the three hours in the afternoon from 2 to 5. Dinner is at 1 and 1.30, and supper at 9.15. They go to bed at 10. In summer they go into the grounds after supper to get some fresh air before going to bed. In winter, on fine moonlight nights, they get permission to slide until bedtime. There is a fine course from the hospital to the gate, and the exercise is most exhilarating—the swift run down the hill through the keen frosty air, then the climb back up the hill pulling the slides behind. A few tumbles are inevitable and only add to the enjoyment, and the nurses come in with rosy cheeks, looking pictures of health. Occasionally the hour off duty has to be taken at night, and then they may go to the rink, which is within a few minutes' walk, and have half-an-hour's skating. The night nurses go on duty at 9 P.M. and come off at 8 A.M., breakfast at 8.15, report at 8.45. In summer they have the evenings off, go to bed at 9 A.M., and are called at 5 P.M. In winter they are off duty in the morning, go to bed at 12, and get up at 8. They get a day off every fortnight; night nurses may sleep out if they wish to do so. On Sundays the time off duty is 7 to 9.45, 9.45 to 12.30, or 5.30 to 9. Roman Catholic nurses are always off duty in the morning, others may have morning or evening as they prefer by mentioning it the night before. A short time ago the hospital was honoured by a visit from Prince Louis of Battenberg. He was accompanied by His Excellency the Governor, who is himself a medical man, and visited every ward in the hospital. Almost every visitor remarks how "nice and fresh the wards are." This is owing to the fine pure air and the healthy surroundings, which conduce much to the success of the operation cases of the hospital.

The patients are visited every week by ladies of the Cowan Mission, which was organised in memory of Miss Cowan, a former matron of the hospital who devoted her whole life to the work, and died in harness. These ladies bring little delicacies to tempt the patients' appetites, or books for them to read, and their visits are greatly enjoyed. It is to their efforts that the convalescent home is due. Here the convalescent patients are sent to make room for the more acute cases, which would otherwise have to be refused for want of room.

### Christmas in the Hospitals.

BY OUR OWN COMMISSIONERS.

(*Concluded from page* 203.)

### KING'S COLLEGE HOSPITAL.

Shortly before midnight on Christmas Eve the nurses and resident staff assembled in the corridors of King's College Hospital, in order to perform their usual ceremony of singing Christmas Day in with carols. As the clocks struck twelve the singing ceased, and the hospital slept once more. On Christmas Day itself there were unwonted privileges to be enjoyed. Visitors were welcomed, the men patients were permitted to smoke, and turkey and plum-puddings were distributed to all the inmates. The usual Christmas services were held in the course of the day, and at five o'clock evensong an anthem and carols were sung, and were much appreciated. The brightly illuminated windows of the hospital, showing up the flowers and decorations which adorned the wards, told to the outside world on December 28 that "King's" was still keeping up its Christmas festivities. Inside the hospital all was bustle and preparation on the part of the resident staff, students, and nurses, who had prepared an elaborate programme for the entertainment of the patients. The decorations were still most festive-looking and gay, many-coloured Chinese lanterns and fairy lamps contributing largely to the picturesqueness of the scene. The patients all wore a look of pleased expectancy, and in their scarlet jackets, and in many cases quaint-looking paper caps from the crackers, all seemed quite in keeping with the happy season. At four o'clock in the topmost ward, Todd's, the proceedings began by a short concert, consisting of songs and instrumental music, and ending by a part-song delivered by a chorus of nurses, which met with much applause. To this succeeded a visit from some very "sleepy minstrels," appropriately attired in bedgowns and nightcaps, bearing each a candlestick in his hand. So overcome with sleep were they apparently that it was with difficulty they roused themselves sufficiently to sing some most amusing songs, which were highly appreciated by their delighted audience. How "Father laid the carpet down the stairs," sung in harmony, was a special favourite, but best of all the patients loved the allusions to "King's," and the statement that after they had been enjoying "turkey, plum-pudding, and spice," they would all have to go back to "plain boiled rice," was hailed with great laughter. When the sleepy minstrels had yawned themselves out of the room there immediately entered a tumultuous troupe of gaily-attired golliwogs, headed by a most facetious policeman, with nose and ears of quite an abnormal length. The policeman's rendering of a song narrating the misadventures of a motor-car trip to Brighton roused great merriment, and after a most comical performance, winding up with a cake-walk, they withdrew to delight the other wards with their pranks and songs. The entertainment lasted over an hour in Todd's Ward, and the performers must have felt somewhat exhausted when their final performance in Fisk Ward was given. Each batch of course passed on to the next ward directly they had played their parts, so that no time was lost. The whole performance reflected the greatest possible credit on all who had worked so hard and energetically to make it the great success it undoubtedly was.

### THE LONDON HOSPITAL.

All the Christmas festivities at the London begin and end on Christmas Day itself. At four o'clock in the morning a band of carol-singers, composed of nurses and the resident staff, ushered in the day. Breakfast over, Father Christmas began to make his rounds attended by seven or eight little girls from the convalescent ward attired as clowns—very necessary helpers, for a long and arduous task lay before Father Christmas. Thanks to the generosity of Mr. Edgar Speyer, every patient in the hospital got a really handsome gift on Christmas, such as cardigan jackets for the men, and warm shawls and petticoats for the women. Many other kind donors sent gifts, and so it came to pass that each one of 719 patients in the London last Christmas Day was rejoicing in a useful present. Careful preparation is made beforehand for their proper distribution. The sister of each ward is asked to send down a list of her patients and their probable requirements. Then a bale of suitable presents is deposited in her care and she has them packed up separately in brown paper. The parcels are next stacked outside the doors of each ward, ready for Father Christmas and his assistants. It took this important personage four hours to complete the rounds of the wards and distribute to the patients personally their Christmas gifts. By that time the dinner-hour had arrived. Roast beef and plum-pudding were provided by the authorities, but in nearly every case the sisters were able to supplement this fare with turkeys sent by many kind friends. There were 79 cooked on Christmas Day, so that there should not have been many patients who did not participate in them. After dinner the men received, not only permission to smoke, but the wherewithal to do so, in the shape of pipes, tobacco, cigars and cigarettes. Then the wards were made ready for the entertainments. A proper stage was erected at one end and the patients' beds were drawn up facing it, side by side, leaving only a narrow gangway in the middle. All visitors and onlookers were required to stand behind the beds, a highly desirable arrangement. From 3.30 to 7 there were concerts every half-hour, given by different troupes of entertainers, twenty-three in all, including every known variety. There was a Punch and Judy, a magician, "Weirda the Mysterious Man in White," there were golliwogs and nigger minstrels. An elaborate programme was made out which showed exactly where every troupe would be performing at every half-hour in the afternoon. The little ones had their Christmas-trees, and those able to be moved were brought into the wards for the entertainments. The wardmaids and scrubbers had a dance in the evening in the large out-patients' hall, and there they too were visited by a good many of the troupes. Mr. E. Magnus gave tobacco and fruit, and many sums of money, including £25 from Mr. L. Rothschild, were contributed for the express purpose of affording the patients of the London Hospital a good time at Christmas.

### St. George's Hospital.

At St. George's Hospital all the wards were transformed for the time being by means of artistic decorations of evergreens, gaily coloured fairy lamps, etc. Special provision was made for the little ones in the shape of Christmas-trees, each ward in which there were children having a tree of its own. On Christmas Day the usual seasonable fare was provided, consisting of roast beef and plum-pudding, and was much enjoyed by all well enough to partake of it. On December 27 the patients had their "Christmas tea," a very popular institution and much looked forward to by the inmates. Tea and coffee, fruit and crackers and other delicacies were supplied for all, and many lady visitors and friends attended the function, bringing with them little gifts for the patients. The toys so generously given by the proprietor of *Truth* were divided amongst the children, to their great delight. One small boy named "Bobbie" had certainly received his fair share, for he lay there, flat on his back, clutching at no fewer than four lovely toys—a "gee-gee," a black dog, a donkey, and a very grotesque policeman-doll, which he informed a visitor confidentially was "Daddy"! On the 29th a conjuring and musical performance was held in the Board-room for the benefit of all those patients who were well enough to be brought down to it. Over 100 were able to be present, many having to be carried down on stretchers or in chairs, and all heartily enjoyed themselves. This month there is to be a tea for the charwomen employed in the hospital and also the annual entertainment for the nursing staff.

### St. Mary's Hospital, Paddington.

Every patient at St. Mary's Hospital, Paddington, received a useful present on Christmas morning. In the children's wards large stockings were hung up filled with good things for the little occupants of every cot. There were three celebrations of the Holy Communion in the chapel, which was tastefully decorated. The anthem "Arise, shine" was well rendered by the choir of nurses. All the male patients who were well enough were allowed to smoke; this privilege was thoroughly appreciated. The Christmas dinner was, as usual, a great success. When sister was not looking the children picked out the currants from their molecule of plum-pudding with their fingers and held them up to show their neighbours what they were up to. The nurses' dinner consisted of turkey, plum-pudding, and a very liberal dessert—provided by a generous friend. At four o'clock the ward-teas commenced, and in the wards where patients were well enough music and recitations were the order of the day until 8 P.M., when "God Save the King" was sung all over the hospital. The wards looked very festive; in Alexandra, patients made all the paper flowers. "Ah," said one poor thing as she lay back on her pillows, "making them flowers just a kept us from fretting over our good men and the children; we were so busy trying to see who would make the most that we all felt better, and now we're proud women to-day; we do feel so grand in our smart jackets, and sister is so proud of us all, bless her." Every ward was given a piano for Christmas and Boxing Day, only to be used from 4 to 8 P.M. The Christmas-tree was dismantled in the Board-room on Wednesday, December 27, at 4 P.M. The dramatic entertainments by the residents in the out-patient hall of the Clarence Wing, December 28 and 29 closed the festivities. The first night was for patients and night nurses and their friends. The second night was for visitors and for the day nursing staff and their friends.

### Hospital for Sick Children, Great Ormond Street.

It would have been difficult to find any happier children in the three kingdoms than the little in-patients at Great Ormond Street Hospital last Thursday afternoon. Turning from the dismal city streets where mist, mud, and a general air of malaise prevailed, into the bright and beautifully-decorated wards, it was hard to realise that it was a world of pain and not an enchanted palace. The chief feature in each ward was the Christmas-tree—such trees, seven in all, their tops almost reaching the ceiling, and wide-spreading branches bending beneath the load of toys—and the lights—hundreds of them, fairy lights, electric and candles—making the frost and tinsel sparkle and shimmer like snow on a moonlight night. Underneath the trees lay mysterious packets, and groups of convalescent children, boys in sailor suits in abated suspense akin to agony, the model of propriety (*pro tem.*), and daughters of Eve, prettily clad in white frocks and stockings, gay sashes, and hair-ribbons, fully conscious of their charms, sat round the fire and speculated as to what Father Christmas would bring them, or stood round the tree in breathless expectation. Other children were on couches and wheel-chairs. Nurses stood round also, with white bundles in their arms which, being interpreted, were babies in soft woollen jackets and embroidered head-flannel, too young for toys, but boldly asserting their right to a certain amount of attention. The toys were really beautiful and of every imaginable description—dolls in various costumes, books, bricks, engines, and others of a mechanical order. The wards rang with laughter.

### CHRISTMAS IN THE HOSPITALS—*continued.*

Grey-haired dignified members of the faculty unbent and smilingly presented the happy, excited little ones with their gifts, which were received with exclamations of delight and round bright eyes of wonder. In Alexandra Ward a little girl was going into raptures over a mechanical rabbit, which wobbled in a ludicrous manner on its hind legs; "You see," she explained with a wise air and firm conviction, "it's going to market." "To buy a fat pig," the onlooker suggested. "Yes, that's it," she added, delighted; "here's the basket." At this moment she was presented with a doll sufficiently imposing to eclipse all other joys, labelled "Queen Maud of Norway in National Costume." For a moment the child was speechless, then she eagerly stretched out both arms, and with the quaintest motherly air in the world took it and pressed it to her. The poor rabbit wobbled gravely on—forgotten—to certain death and destruction had not a friendly hand stayed his progress. One sweet baby, aged about two years, had discarded a large white woolly lamb, and, oblivious to all around, was lost in admiration of the intricate beauty of a spray of mimosa, to which it apparently had a good deal to say in its own way. In Helena Ward there stood by the fire an old wooden cradle on rockers; from beneath snowy sheets and warm blankets two tiny pink fists protruded making a quaint and home-like element. The wards were open from 4 to 6 P.M., and visitors and friends were allowed to wander about as they wished. From time to time strains of music and gramophone records mingled with the sounds of revelry—a tiny bandaged head wagging time and small feet gaily tapping. The usual distribution of toys in memory of Corney Grain took place. Before leaving for India the Princess of Wales sent toys and dolls for the children. Books had been sent from "Punch" and Mr. William Younger, M.P. Sir Tollemache Sinclair and Mr. James Jay, of Potters Bar, both sent gramophones. More toys came from Sir Squire and Lady Bancroft, and two ladies, resident in Moscow, sent dolls. To complete distant memories a case especially sent from New Zealand arrived in the out-patient department on Thursday morning. Many clever heads and hands must have been hard at work in the wards to produce such results as were in evidence : in each ward some special colour predominated—the numerous lamp-shades and fairy lights in delicate tints of blue, red, mauve, and green setting off the whole. Tulips, violets, carnations, lilies-of-the-valley, daffodils, etc., filled the air with fragrance. Holly, smilax, and ivy were not lacking. In Clarence Ward over a table tastefully arranged with mimosa hung a cage in which a canary was singing his heart out for very joy. These latter things were noted and wondered over by one or two pale and sad little faces, lying still, too weak to be boisterously happy, but doubtless helped by them to forget their pain.

### Mount Vernon Hospital, Northwood.

Christmas at the beautiful country branch of the Mount Vernon Hospital at Northwood has been celebrated with old-fashioned goodwill and merriment. Kind friends from far and near, including The Hospital, have united to fitly mark this joyous festival. Numerous and varied features characterised the programme. The Sunday Christmas Eve service in the picturesque and tastefully decorated hospital chapel was made bright by carols and an anthem rendered by the choir composed of the resident staff. Many relatives of the patients attended and were afterwards entertained at tea. In the evening the hospital choir played the part of waits, and with lanterns serenaded the patients with old-time carols. For Christmas Day, Santa Claus had bountifully provided acceptable gifts which were highly appreciated. Every part of the building was bright with decorations. The wards were gay with flowers and evergreens and a large Christmas-tree, brightly lit and heavily laden, adorned the dining-hall. An old-fashioned Christmas dinner was provided, of which most of the patients were able to partake. After tea a unique entertainment was given by the patients and staff and included an amusing presentation of Mrs. Jarley's waxworks, a coon sketch and songs, and a performance of Romberg's Toy Symphony. After this, Father Christmas arrived to distribute his souvenirs. On Boxing Day the medical and nursing staff and their friends dined together, after which the matron and sisters afforded a delightful surprise by the performance of five dainty old-world scenes from Mrs. Gaskell's ever-appreciated "Cranford." This proved so attractive that it was repeated on two subsequent evenings for the benefit of the patients and the large domestic staff. The members of the latter, each tastefully dressed in fancy costume, dined together and afterwards contributed a delightful programme of songs and sketches. A long-to-be-remembered week closed with an amusing entertainment by a conjuror and ventriloquist, kindly provided by the chairman of the hospital.

### East London Hospital for Children.

At the East London Hospital for Children Christmas is celebrated in quite the most orthodox style, and with strict adherence to all the time-honoured rites. On Christmas Day each little inmate discovered a stocking in its cot, which stocking contained an orange, some sweets, and some other delicacy. The wards were made as like fairyland as skilful hands, quantities of lovely flowers, and trailing evergreens could make them. Then Father Christmas went round with gifts of most desirable toys for everyone. In the afternoon of Christmas Day there were games for those who were able to play them. But all this was only the prelude to the great entertainment which took place on December 29. About three o'clock nurses, doctors, and kind helpers began the work of carrying the little ones down to the out-patients' hall. Here they were laid on mattresses in a large semicircle facing two stately and heavy-laden Christmas-trees, with between them the gaily-painted booth of a marionette show. The convalescents were ranged on forms behind the mattresses, and behind them again were tables with more mattresses and more eager little spectators. The very tiny ones were in great request among the lady visitors. It was a very pretty if pathetic sight to see all that gathering of little sufferers, some rolled in blankets, all spotlessly clean, in their bright scarlet jackets, most of them wearing gay paper caps. The show itself was a most laughable performance from beginning to end, and it was delightful to watch the little pale faces beaming with merriment and to hear the laughter break out, even if it was rather weak and thin. Next came the lighting-up of the two great trees, and the nurses all swarmed round them collecting the presents for their own special charges. Such beautiful presents they were, too : not little penny toys, but well-dressed dolls, puzzles, books, and—alas for the poor nurses!—many instruments of music. All, of course, were not able to come down to the entertainment, but a visit to the wards while the Christmas-trees were still in full swing showed that those left behind had not been forgotten. They did not seem in the least to consider themselves objects of pity, as they lay in their almost deserted wards. For instance, Nellie, who lay clutching a very smart dolly and a yellow duckling, looked the picture of joy. Dolly was "a gweat weight" she remarked, but this she seemed to consider a distinct advantage. She was rather hazy as to what a duck was in real life, never having probably encountered one, but she was very insistent that birds "often fly over our loof." It is comforting to think that when the poor little mites go back to their homes they will have such bright memories to look back upon of the time they spent Christmas in hospital.

St. Marylebone Infirmary.

"Inspection Day" last week at St. Marylebone Infirmary was a greater success than ever, between five and six hundred guests responding in person to the invitation of the Guardians. Conspicuous among these were some well-known figures in Marylebone: Mr. White, Chairman of the Board; Alderman Dennis, the Mayor of Marylebone; Miss Vincent, the former matron; and Miss Moriarty, from Isleworth. Dr. Brown, Chairman of the Committee, received the visitors, along with the matron, Miss Ramsden, who, in her present position and her former one of assistant matron, has worked for fifteen years in the Infirmary. All the Committee were present, and most of the Guardians. Tea and coffee, and cakes of many varieties were served in the Board-room, after which the constantly renewed stream of guests, escorted by sisters, nurses and doctors, passed on to the gaily decorated wards, bright with hundreds of Chinese lanterns and fairy lamps shining on the glistening evergreens and scarlet holly-berries. At the top of one staircase, leading to the men's phthisis wards, an Arab encampment had been erected, the camel, made out of brown paper and cloth, testifying to the ingenuity of some of the patients. Tent, sand and straw were all complete, and the figure of the Arab, draped in white, with painted mask, was exceedingly good. Near by was a similarly constructed figure of a nigger, banjo on lap, and hung on his chair was a large placard with some amusing lines written by a patient. Another form of entertainment was the carol-singing by the nurses, who made the round of the twenty-four wards, accompanied on the harmonium by the organist, Mr. Wilson, who had carefully trained them for the performance. Great interest was shown in the huge laundry, which contains some of the most-approved modern machinery. All the nurses, down to the newest probationer, were allowed to invite their friends, and several confidential little groups had snugly ensconced themselves in various corners of the Nurses' Home, newly decorated this year in such pretty colours. This was the last day of the season's festivities. The children had had their Christmas-tree on Tuesday, and after the visitors had gone all the decorations were to be ruthlessly torn down, lest "microbes" should find a home in the dust which inevitably collects on the manifold surfaces. The only door which was not thrown open wide to all who wished to enter was that of the operating theatre, the pride and delight of Dr. Lunn's heart, recently built and fitted up with all the newest appliances. It was kept jealously guarded, and only a few privileged ones were allowed to intrude their dusty garments within the sacred precincts of Asepsis.

City of London Lying-in Hospital.

Christmas Day celebrations at the City of London Lying-in Hospital were commenced early in the morning. At 6 A.M. the nurses formed a long procession, carrying lighted Chinese lanterns and singing Christmas carols and hymns in every ward. Father Christmas visited each patient, and after wishing them a very happy Christmas he opened his huge sack and took from it a very large bundle for every mother, containing useful clothing for herself and infant. Later on the matron presented a robe to each of the Christmas babies. Afterwards the patients enjoyed an excellent dinner, consisting of boiled chicken, followed by Christmas pudding and dessert. In the afternoon an entertainment was given in one of the wards by the nurses, and all patients able to be up were present; they greatly enjoyed the music and nursery rhymes which were performed in character, the nurses having spared no pains in preparing beforehand. Tea and cake were handed round to the patients, when Father Christmas appeared once more, and each mother had again a very useful present. Bed followed, and after the lights were turned down the nurses formed in procession with lighted Chinese lanterns, and sang carols in each ward. After the patients had retired for the night the nurses had their turn, and singing, dancing, music, and recitations were carried on until midnight, when "Auld Lang Syne" was heartily sung, and the nurses scampered off to bed.

Paddington Green Children's Hospital.

Santa Claus paid the children of the Paddington Green Hospital a visit on Christmas Day itself, and as usual received a most cordial welcome. His first act, before the eyes of the little ones were open, was to fill the stockings hanging on their beds. Later on the delighted children found that he had hung upon the lofty Christmas-tree, shining with 100 electric lights, a gift for everyone, even for those who were not well enough to leave their beds. In addition to the excitement of Christmas fare and appropriate presents, the little convalescents were entertained in the surgical ward with a series of pictures—some pathetic, some comic—thrown on to the screen by one of the medical men. All this gladdening of the small patients brought additional pleasure to those connected with the hospital itself, because all the expenses were defrayed by private friends, not one farthing being taken from the already over-burdened hospital funds. The decorations, which were particularly good, were also contributed.

Hospital for Epilepsy and Paralysis.

A very enjoyable Christmas was passed at the Hospital for Epilepsy and Paralysis, Maida Vale. On Boxing Day the resident medical officer, Dr. Frederic Barker, and the nursing staff gave a greatly appreciated entertainment to the patients and their friends, which included songs and choruses (in costume) from "The Geisha" and "The Country Girl," while on the following day another entertainment offered by Captain Ellis, a member of the Committee, was much enjoyed. At the end of the former evening Dr. Barker (as the "Rajah of Bhong") brought down the house by his (Cingalese) parody (with apologies to Messrs. Lionel Monckton and Rutland Barrington) of which the burden was "There's nothing much more to say."

North Eastern Hospital for Children.

The annual children's Christmas treat for in-patients at the North Eastern Hospital for Children, Hackney Road, Bethnal Green, took place on Thursday, December 28, and was greatly enjoyed by the children, as well as by the many friends of the institution who assembled to assist in the distribution of gifts and to see the wards on the occasion. Four large Christmas-trees were placed in different wards, and each of the nine wards in the hospital had its own scheme of simple but effective decoration. The New Year and the Old Year, represented respectively by a little convalescent girl patient and one of the house surgeons, made a tour of the wards, and handed a toy to each patient. Three "Punch and Judy" shows were given in some of the wards, where special arrangements had been made for the purpose.

Royal Victoria Hospital, Belfast.

Christmas was looked forward to at the Royal Victoria Hospital, Belfast, with the usual sense of expectancy, and as the day drew nearer the excitement increased. On the Friday and Saturday beforehand every spare minute was occupied by the sisters and nurses assisted by the convalescent patients in decorating the wards, with the usual competition as to whose ward would look the prettiest. However, in the long run, each ward was very gay and pretty in its own way, and the workers felt that they were repaid for the trouble they had taken. The decorations consisted chiefly of evergreens arranged in various ways; the electric lamps were draped with paper shades, each ward having its own special colours according to taste. The morning of Christmas Day was spent in preparing for what was to the patients the chief event of the day—the dinner. The tables in the wards were spread and decorated daintily with flowers and plants, sweets, fruit, etc., by the nurses; the patients watching all operations with interest. At 2 P.M. dinner arrived, the staff physicians and surgeons also arrived, accompanied by friends, to carve and serve it. All the patients who were able to partake of the meal seemed to enjoy their treat heartily. After dinner the wards began to fill up with the patients' friends, who were allowed the privilege of visiting the invalids as an extra treat on Christmas Day. At 4.30 P.M. an "at home" was held by the nursing staff, each having invited beforehand one or two of her own special friends. One of the empty wards —several being still empty owing to lack of funds—was converted into a reception-room and decorated prettily for the occasion. At 7.30 P.M. a musical entertainment was given in the large waiting-hall of the Extern Department, which was decorated with evergreens and pretty lamp-shades. The concert, which consisted of a varied programme, was given by friends of the staff who kindly lent their assistance for the evening. All the patients who were well enough were taken to the concert-room. The day at last came to an end, but will probably be looked back upon with pleasure for many days to come.

### A CHRISTMAS NUMBER WRITTEN BY NURSES.

*** It is intended to make the feature of our Christmas Number of 1906 a series of incidents occurring at the Christmas season just past, and we therefore invite nurses engaged in general or special hospitals, in Poor-law infirmaries, in mental institutions, and in district or private work, whether at home, in the Colonies, or abroad, to send in contributions, varying from 500 to 1,000 words, not later than March 31 next. Original photographs illustrating the articles will be very acceptable, and these and the literary contributions used will, of course, be paid for at the usual rate. Preference will naturally be given to the most carefully and brightly written accounts, but incidents of exceptional interest will not be passed over because the writer is deficient in literary style and composition. The contributions should in all cases be addressed to the Editor of the Nursing Section of THE HOSPITAL, and marked outside "Christmas Incidents."

### Presence of Mind.

### A PRACTICAL ILLUSTRATION.

THE life of a patient has often been saved by the presence of mind of a nurse. The following is a true incident:

A charge-nurse on night duty alone in a men's ward had several very bad cases. One of them had shown signs of dementia during the day, so a porter was told off to sit by him during the night. The patient was a man of more than sixty, and had developed a great liking for the nurse. Many a time he said to her—not at all disrespectfully, but like an old man who rambles on hardly knowing what he is saying—"Now, nurse, you might give me just one kiss before I die." And the nurse invariably replied: "Don't talk nonsense, daddy; I only kiss my relations." On going out to get her breakfast on the night in question the nurse enjoined the porter to be very careful to keep a good watch on the man, because she was sure that he was only pretending to be asleep, and the porter had promised to be careful. But the nurse had only been gone two or three minutes when something prompted her to go into the ward again. To her horror she saw poor old daddy standing up on one of the high window-sills, just ready to jump out. For a moment her heart seemed to cease to beat, but she managed to control her voice enough to say: "Daddy, what are you doing?" "Just going to throw myself out, my dear; nobody wants me," was the reply. Like a flash of lightning an inspiration came into the nurse's mind. "Well, come and give me that kiss first." Down jumped the poor old man at once, quickly to be overpowered by the porter and nurse, who strapped him down in bed. When he was safely there he said pathetically: "Ah, my dear, I never thought you would serve me like this." But the nurse was too glad to know she had saved her patient's life to mind even the reproaches.

### Everybody's Opinion.

[Correspondence on all subjects is invited, but we cannot in any way be responsible for the opinions expressed by our correspondents. No communication can be entertained if the name and address of the correspondent are not given as a guarantee of good faith, but not necessarily for publication. All correspondents should write on one side of the paper only.]

### A ROYAL COLLEGE FOR NURSES.

DR. JOSIAH OLDFIELD writes: In your criticism of my article on the above subject you have—inadvertently, I am sure—made me talk much nonsense. I should no more suggest such a degree as "Bachelor of Nurses" or "Mistress of Nurses" than I should think of such degrees as "Bachelor of Physicians" or "Master of Lawyers." The remedy which I suggest for the present chaos is the introduction of a definite diploma of a definite value, and open to all to enter for—and this diploma will be obtained in a somewhat similar way to that in which diplomas given in Arts, Law, Music, Medicine, etc., are obtained. Nursing will then rank as a similar profession, and the degrees would be similar—namely, B.N. (Bachelor of Nursing), and M.N. (Mistress of Nursing).

[We regret that, owing to a mistake in copying, an error was made in quoting Dr. Oldfield's words.—ED. THE HOSPITAL.]

### THE NURSE'S CLINICS.

"M. H." writes: As a general nurse and midwife, may I say that I am very pleased to see the questions on puerperal nursing, infant feeding, etc., in the NURSING MIRROR, and I should like to know if you can tell me the effect of opium on the pregnant woman before delivery. Myself I do not believe in giving sedatives before, but as I know a doctor who does so, I should like to have your opinion. I may say, as a fully certified nurse, I know the action of the drug in a case of need. In a word I want to know if it does the patient good or harm. I should like to say in regard to corrosive sublimate in my midwifery training we used $\frac{1}{2000}$ from the birth always with good results. Sometimes nitrate of silver in fluid form was necessary, though none but matron or sister in charge were allowed to use that. In my general training I frequently syringed the eyes with the last named with very good results. I have had the care of children almost blind through neglect at birth. But for sure and safe measures nothing beats boracic lotion. Though I always use $\frac{1}{2000}$ cor. sub. I notice you are frequently asked for homes for invalids or chronic patients, would you kindly give my name to some of your inquirers?

[No general rule can be laid down with regard to the use of sedatives in pregnancy or any other condition. Their probable value can be estimated only by the patient's medical attendant, and they should never be used except by his orders. Boracic acid solutions, though harmless, are of little value as disinfectants; and therefore are neither sure nor safe in cases where energetic measures are required. We cannot give names of private nurses to inquirers.—ED. THE HOSPITAL.]

"OUR CHRISTMAS PARTY."

THE MATRON OF THE GARFORTH FEVER HOSPITAL, YORKSHIRE, writes : As a subscriber for years to your valuable paper, I was reading an incident of a nurse's life about her Christmas party in hospital, and I thought I should like to describe our little isolation hospital at Christmas, that the outside world may see how happy children can be at Christmas, even if isolated from home. The hospital holds forty patients when full. The staff consists of matron, three nurses, wardmaid, and caretakers, who act as cook and porter. Quite a month beforehand we made tissue decorations in all our spare moments. Two of our little girls, only four years old, were taught to put the links of the chains together, and made many yards. A male scarlet patient painted numerous jam jars bright red, which looked lovely with white and coloured flowers in them when placed on the window-sills. About a week before the 25th decorating commenced in real earnest, everyone helping. Large mottoes were erected over the doors, and the beams were covered with holly. All was well finished by Saturday night. Christmas Eve being on Sunday was of course kept rather quietly. Carols, sung by the nurses and by sixteen scarlet patients, were very much enjoyed. After the children had retired for the night, and an interval of an hour, the matron and nurses went stealthily round filling the stockings with various toys and other things. Early next morning the shout was raised, "Santa Claus has been and filled our stockings," and the whole ward was soon uproariously happy. Breakfast was served about 8 A.M. After breakfast was finished the children had plenty to do showing each other the toys and crackers brought by Santa Claus, and also in peeping at their friends, who were allowed as a treat to look through the windows at the patients and to see the Christmas-tree, which was loaded with presents, etc. The work of the wards was soon accomplished, everybody intending to finish quickly. Dinner time was arranged for 12 P.M. The table was prettily set, and flowers arranged up the centre of a long table. The children allowed out of bed were on each side, the matron and three nurses attending to their wants. A huge piece of beef, with Yorkshire pudding and vegetables, graced one end of the board, followed up by plum-pudding and sweet sauce. Those very ill were only allowed the special diet ordered by the doctor. After dinner had been cleared away games were indulged in, and in the middle of the afternoon fruit was given out. Tea was prepared about 4 P.M., with cake, mince-pies, and sponge-cakes, and plain biscuits for the special invalids. As soon as tea was disposed of the place was made tidy to await Father Christmas, who arrived about 5 P.M., all the blinds being drawn up to allow the children to see him pass the window, dressed in a scarlet cloak, with red hat edged with white fur, a white beard, a thick stick and a piece of laurel in his hand. He slowly wound his way past the scarlet block across to the typhoid block, which he entered with his heavy-ladened sack on his back. He had to go through very quietly, as there were two bad typhoid cases, and gave a male patient, aged 26 years, a game, and a boy of 13 a mechanical toy. As he slowly came back past the scarlet block all the children were anxiously waiting for him to enter, but when he had passed the last window they exclaimed with one voice, "Father Christmas has gone, and is not coming here." This was done as a joke, as he was entering by another door. "A Merry Christmas, children," he said in a deep voice as he came through the door, and then reaching the centre of the room he stood and opened his sack. There was a toy for everyone—dolls for the girls, mechanical toys for the boys, and stuffed animals for the wee ones. At last all was over; Father Christmas said "Good-night," the children had milk, cake, and biscuits, and listened to more carols until 8 P.M., when all patients were put to bed and lights lowered on a very happy family. The tired but happy nurses then at last sat down to supper, having in their turn enjoyed the making of so many happy. I would like to add that the Council kindly forwarded all the toys, and thus materially lessened the expenses for the nurses.

### Appointments.

CANTERBURY NURSES' INSTITUTE.—Miss A. A. Gwyn has been appointed lady superintendent. She has been Matron of the Royal Hants County Hospital, Winchester, and Lady Superintendent of the County Hospital, York.

GLOUCESTER GENERAL INFIRMARY.—Miss A. Hamilton has been appointed night superintendent. She was trained at the General and Dispensary Hospital, Bury, where she was afterwards appointed night sister. She has since done private nursing, and has been staff nurse at the Kendal Hospital, Westmorland, and ward sister at the Western District Hospital, Glasgow.

HAMMERSMITH INFIRMARY, WORMWOOD SCRUBS.—Miss Eve Ward has been appointed matron. She was trained at the Hope Hospital, Salford, where she afterwards became successively sister of the ophthalmic wards of the male pavilion, sister of the operating theatre, and temporarily superintendent of night nurses. She has since been assistant matron of St. Luke's Hospital, Halifax.

KING WILLIAM'S COLLEGE, CASTLETOWN, ISLE OF MAN.—Miss Ethel Shaw has been appointed charge nurse. She was trained at Shaw Street Hospital, Liverpool, and was subsequently on the private nursing staff of that same institution. She has since been at Sir William Sinclair's Surgical Home, Manchester, has done private work in South Wales, and has been on the staff of a nursing home at Chester.

NOBLE'S HOSPITAL, ISLE OF MAN.—Miss Mary Fisher has been appointed charge nurse. She was trained at Guy's Hospital, London, where she was afterwards staff nurse and theatre nurse. She has also done nursing in South Africa as a sister of the Army Nursing Service Reserve, and has since been charge nurse at Aberdare Hospital.

QUEEN'S NURSES' HOME, GRIMSBY.—Miss Katharine Lunn has been appointed superintendent. She was trained at the Salop Infirmary, Shrewsbury, and has since been ward sister at Leith Public Health Hospital, Queen's nurse, and subsequently assistant superintendent of the Northampton Town and County Nursing Institute. She is registered under the Central Midwives Board.

ST. HELEN'S BOROUGH SANATORIUM.—Miss A. Pearson has been appointed staff nurse. She was trained at Crumpsall Union Infirmary, Manchester, and the General Hospital, Loughborough, where she has since been staff nurse.

WHITEHAVEN INFIRMARY, WEST CUMBERLAND.—Miss F. McGillivray has been appointed night sister. She was trained at the Holborn Infirmary, London, where she was afterwards charge nurse and night superintendent. She has since done private nursing, been superintendent at the Walsingham Union, and ward sister at the Western District Hospital, Glasgow.

### Our Christmas Distribution.

THE matron of the Royal Free Hospital, Gray's Inn Road, writes: "I beg to acknowledge with the most grateful thanks the parcel of clothing sent to us at Christmas. They were such very useful articles, and the greatest help to us in providing gifts for our people."

### "The Hospital" Convalescent Fund.

THE Hon. Secretary begs to acknowledge with thanks the receipt of 2s. 6d. from Miss E. Jones, per the Royal National Pension Fund, and 8s. 6d. from Nurse Ruel, a kind contributor who utilises every opportunity of helping the fund.

### Notes and Queries.

**REGULATIONS.**

**The Editor is always willing to answer in this column, without any fee, all reasonable questions, as soon as possible.**

**But the following rules must be carefully observed.**

1. **Every communication must be accompanied by the name and address of the writer.**
2. **The question must always bear upon nursing, directly or indirectly.**

**If an answer is required by letter a fee of half-a-crown must be enclosed with the note containing the inquiry.**

*Pension or Insurance.*

(96) Will you tell me if it would be a greater advantage for nurses individually or collectively to join a well-recognised insurance company or the Royal National Pension Fund? They are cared for in the institute during sickness; the object of the insurance is to acquire for them an old-age pension. They would receive assistance from a general fund. —*H. M. S.*

The advantages of the Royal National Pension Fund over ordinary insurance companies are very substantial.

*Lectures to Women.*

(97) I shall be much obliged if one of your readers who may probably have given lectures on nursing to women, could tell me of suitable subjects to take, or a book that would assist me. I am a three-years' trained nurse, and hold L.O.S. certificate.—*Daisy.*

"Lectures on Nursing of the Poor," by A District Nurse, price 1s. 6d., published by The Scientific Press, Limited, 29 Southampton Street, Strand, W.C.

*Nursery Nurse.*

(98) Will you kindly give me your advice? I wish to become a nursery nurse. Can you tell me of any institution where I can get training? The Princess Christian and Norland Institutes are much too expensive.—*E. T.*

Write to the Liverpool Ladies' Sanitary Association, 8 Sandon Terrace, Liverpool; and the Convalescent or Permanent Home, Uplands, Loughton, Essex; or the Matron, The Home, The Wrythe, Carshalton.

*Mental.*

(99) Please inform me if you know of any home or hospital where a young girl could be admitted who is slightly deficient mentally, and also requires medical treatment.—*N. B.*

Write to the Secretary of the National Training Home for the Feeble-minded, 36 King William Street, London, E.C

*Ships' Nurses, etc.*

(100) Can you tell me if there is a line of ships which engage trained nurses independent of the stewardess, and, if so, where could I get full particulars? Also, can you tell me if the Colonial Nursing Association engages nurses and sends them abroad?—*A Reader.*

The Royal Mail Steam Packet Company, Southampton, employ trained nurses. For information write to the manager. Yes, the Colonial Nursing Association engages nurses and sends them to home colonies. For full particulars write to the Hon. Secretary, Imperial Institute, S.W.

(101) Will you kindly give me the names and addresses of the shipping firms that employ trained nurses on their boats as stewardesses or otherwise?—*Sailor.*

See reply above. But so far, we believe, only trained nurses who are prepared to act as stewardesses have been engaged.

*Nursing in the Colonies.*

(96) I should be glad to have the address of the Colonial Nursing Association, or to know where I could write to for nursing abroad? Having no home ties, I should be willing to go to any part of the world where nurses are most urgently required.—*A. N. F.*

Write to the Honorary Secretary, Colonial Nursing Association, Imperial Institute, S.W.

*Title.*

(104) Will you kindly tell me how it is that in some Union hospitals the head is called a superintendent nurse, whilst in others she is called a matron? I have often considered this subject because I find that both are trained nurses and both women have also the maternity diploma.—*District Nurse.*

It is merely a question of custom or precedent in particular Poor-law institutions.

**Handbooks for Nurses.**

| | Post Free. |
|---|---|
| "How to Become a Nurse: How and Where to Train." | 2s. 4d. |
| "Nursing: its Theory and Practice." (Lewis.) ... | 3s. 6d. |
| "Nurses' Pronouncing Dictionary of Medical Terms." | 2s. 6d. |
| "Complete Handbook of Midwifery." (Watson.) ... | 6s. 4d. |
| "Preparation for Operation in Private Houses." ... | 0s. 6d. |

Of all booksellers or of the Scientific Press, Limited, 28 & 29 Southampton Street, Strand, London, W.C.

### For Reading to the Sick.

"FEAR NOT, FOR I AM WITH THEE."

WHEN clouds awoke by sorrow's wand
  Come o'er the soul in heaviness,
Sweet is the thought of heaven beyond,
  A cave of holy quietness;
Like day beneath the waters seen,
Housed in a deep and blue serene,
  A strange unearthly deep repose,
'Mid hanging rocks all calmly laid,
But touched not by their darkening shade,
  The towers of heaven beyond earth's woes.

O blessed Lord, the thought of Thee,
  When clouds our fairer vision mar;
When we are not where we would be,
  And dearest friends are set afar;
The thought that 'tis Thy ruling will,
The thought that Thou art with us still,
  Nearer than ear or eye can know,
Art with us still in life or death,
In blooming life or failing breath,
  'Tis all of heaven we need below.

*Isaac Williams.*

. . . You say that your sun has gone down while it is yet day; and that your path looks bleak and dreary in the gathering twilight. I know it, my friend; I know that the brightness has vanished from your life, and that from henceforth you must endure hardness even unto the end.

But take courage; advance in perfect faith. Mercies you do not dream of now, will be strewn around your footsteps. Powers which till now have lain as sleeping shadows within you, will awake to life; powers of faith, of hope, of love; and of that perfect patience which will enable you to lift your streaming eyes to heaven, and say: "Lord, I am Thine; do with me what Thou wilt; strip me of all earthly coverings; only save my soul alive." Then let the shades of evening fall; let your path be dark and desolate; but in the surrounding stillness you will hear voices from the everlasting hills, and the sound as of the waving of angels' wings around you. One also, mightier than the angels, will make His Presence felt, and as you place your trembling hand in His, and cry, "Lord, guide me, for I cannot see," there will descend a stream of light upon your darkening path, and peace so perfect, that with songs of praise and of thanksgiving you will pursue your way, willing to wait, willing to endure, willing to do all things, and to suffer all things, for His dear sake who is leading you through the valley of the shadow to the fountain of living water and the land of everlasting joy.

*Anon.*

Lead me now and always
  Even to the last,
Till the way is ended
  And the darkness past;

Till I reach the glory
  I was born to share,
This its crown and centre,
  That my Lord is there.

*C. M. Noel.*

## Figures and Tables

**Figure f1:**
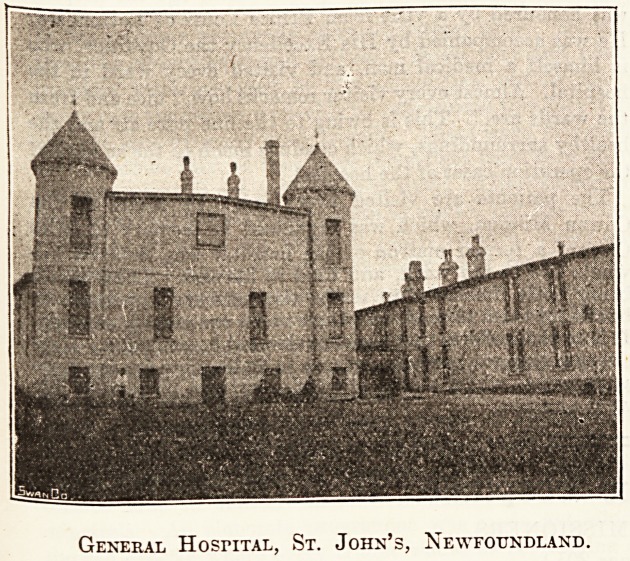


**Figure f2:**
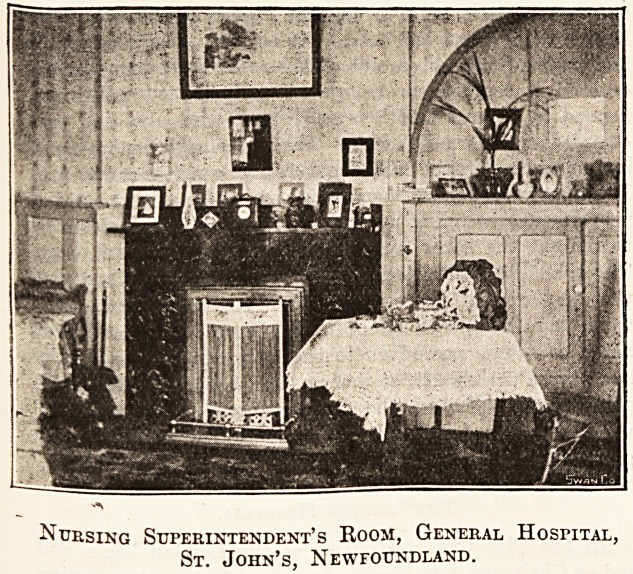


**Figure f3:**
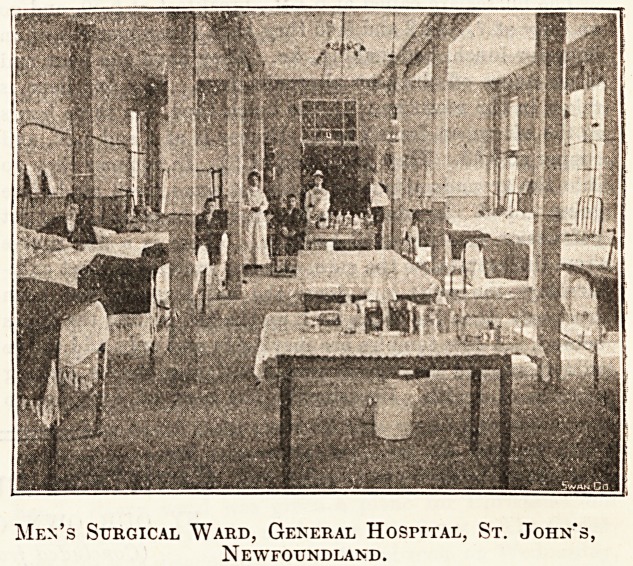


**Figure f4:**